# Gamma-Aminobutyric Acid Increases Erythropoietin by Activation of Citrate Cycle and Stimulation of Hypoxia-Inducible Factors Expression in Rats

**DOI:** 10.3390/biom10040595

**Published:** 2020-04-12

**Authors:** Keun-Tae Park, Jong-Kwon Han, Seong Jin Kim, Young-Hee Lim

**Affiliations:** 1 Research and Development Center, Milae Bioresources Co., Ltd., Seoul 05836, Korea; cerex@naver.com (K.-T.P.); jkhan@milaebio.com (J.-K.H.); sjkim@milaebio.com (S.J.K.); 2 Department of Integrated Biomedical and Life Sciences, College of Health Science, Korea University, Seoul 02841, Korea; 3 Department of Public Health Science (BK21 PLUS Program), Graduate School, Korea University, Seoul 02841, Korea; 4 Department of Laboratory Medicine, Korea University Guro Hospital, Seoul 08308, Korea

**Keywords:** gamma-aminobutyric acid, erythropoietin, erythropoietin receptor, hypoxia-inducible factor, anemia

## Abstract

Erythropoietin (EPO) is the primary regulator of erythropoiesis in the mammalian fetus and adult. Deficiency of EPO induces anemia. In this study, we investigated the effect of gamma-aminobutyric acid (GABA) on serum EPO levels and erythropoiesis in rats. Expression levels of *Epo*-related genes were measured by quantitative real-time PCR (qPCR) and expression of Epo and Epo receptor (Epor) proteins were measured by immunohistochemistry. The gene and protein expression profiles of kidney tissue in GABA-treated rats were evaluated by ribonucleic acid (RNA) sequencing and two-dimensional electrophoresis (2-DE), respectively. GABA significantly increased serum EPO levels and expression levels of Epo and Epor. GABA increased expression levels of hypoxia-inducible factor (Hif)-1 and Hif-2. Seven proteins with expression levels showing >2-fold change were identified by 2-DE followed by MALDI-TOF MS in GABA-treated rat kidney. The top KEGG pathway from the identified proteins was the tricarboxylic acid cycle, and nicotinamide adenine dinucleotide (NADH) dehydrogenase, succinate dehydrogenase, and isocitrate dehydrogenase were identified as key proteins. GABA treatment significantly increased ATP levels and NADH dehydrogenase activity in a dose-dependent manner. In conclusion, GABA shows a new physiological role in EPO production, and it can thus can contribute to the prevention of anemia when used alone or in combination with other anemia treating drugs.

## 1. Introduction

Gamma-aminobutyric acid (GABA) is a ubiquitous non-protein amino acid and its presence has been shown in bacteria, plants, and vertebrates [1,2,3,4]. It is produced by decarboxylation of glutamate by the enzyme glutamate decarboxylase and is metabolized by transamination via the catalyzing effects of GABA transaminase to yield succinic semialdehyde or succinic acid [5,6]. GABA is defined as an inhibitory neurotransmitter in the central nervous system [7] and has various positive effects on mammalian physiology, including reducing blood pressure, relieving anxiety, and enhancing immunity [8,9,10,11]. 

The hormone erythropoietin (EPO) stimulates erythropoiesis, namely the process of red blood cell (RBC) production in the bone marrow [12]. EPO is a hematopoietic growth factor that generally supports proliferation and differentiation of erythroid cells through interaction with the EPO receptor (EPOR) [13,14]. The liver is the main organ of EPO production in the fetus, while the kidney is the predominant organ for EPO production in adults [15]. Approximately 90% of systemic EPO in adults is produced by peritubular interstitial fibroblasts in the renal cortex and outer medulla of renal tissue [16]. *Epo* gene expression in response to reduced oxygen tension is controlled by the heterodimeric transcription factors, hypoxia-inducible factor (Hif), specifically Hif-1α and Hif-2α. Hif-2α has a more critical effect on *Epo* expression than Hif-1α under physiological and hypoxic conditions in adults. Moreover, Hif-1α and Hif-2α have different physiological roles: for example, renal cancer cell growth is retarded and enhanced by Hif-1α and Hif-2α, respectively [17]. 

Anemia with chronic kidney disease shows decreased production of EPO [18]. Diseased kidneys do not release sufficient amounts of EPO, which may consequently lead to anemia, which is universal in end-stage renal disease [19]. Exogenous recombinant human EPO is widely used for the treatment of anemia and generally has a good safety profile. Some potential acute side effects include transient flu-like symptoms, rash at the injection region, and headache, which are normally reported as mild and disappear within a few hours. However, long periods of recombinant EPO treatment can result in cardiovascular diseases including hypertension, polycythemia, stroke, and seizures [20,21,22], with hypertension being the most common [23]. Recombinant human EPO treatment for managing anemia can therefore be costly, have undesired side effects, and be painful to the patients. 

Endogenous EPO produced in the body has a positive effect on various diseases such as nerve cell protection, chronic ocular hypertension, and juvenile chronic arthritis [24,25]. EPO levels in diabetic chronic kidney disease also serve as an indicator for predicting mortality, and increasing endogenous EPO levels is effective in the treatment of anemia in patients with chronic kidney disease [26]. Furthermore, EPO levels predict the mortality rate of patients with heart failure, and consistently elevated EPO levels have independent prognostic value [27,28]. In our preliminary experiment, we found that GABA increases the absorption of organic iron in mice with iron deficiency anemia. Co-treatment by GABA and piperine induces *Epo* and *Epor* genes in kidney cells through p38/c-JUN N-terminal kinase (JNK) mitogen-activated protein kinase (MAPK) activation [29]. Thus, we hypothesized that GABA treatment may improve anemia and increases endogenous EPO levels that stimulates erythropoiesis. To confirm the hypothesis, we investigated the effect of GABA administration on EPO levels and hematological parameter. We investigated expression levels of genes involved in erythropoiesis in rats, and identified proteins involved in EPO production by GABA. We also analyzed a network of identified proteins and found that GABA induces a hypoxic environment, which activates Hif resulted in the increase of EPO production.

## 2. Materials and Methods

### 2.1. Animals

Sprague–Dawley rats (five-week-old, male) were purchased from Koatech (Pyungtaek, Korea). Animals were acclimated for one week under 12 h light and 12 h dark conditions in a room at constant temperature (20 ± 2 °C) and humidity (50 ± 5%). Animals were fed a standard diet (Harlan Diet 2018S; Harlan Laboratories, Madison, WI, USA) with drinking water provided freely. The drinking water was changed every day. All experimental procedures were approved by the Korea University Institutional Animal Care and Use Committee (Approval No.: KUIACUC-2016-148), and animals were maintained in accordance with the Guide for the Care and Use of Laboratory Animals (NIH Publication No. 85-23, 1996). After a week of adaptation, rats were randomly divided into 4 groups (*n* = 8 per group), and the groups treated with 50, 100, or 200 ppm GABA for 3 weeks (Sigma-Aldrich, St. Louis, MO, USA), designated G50, G100, and G200, respectively. GABA was supplied via water. Clinical signs were monitored every 12 h. 

### 2.2. Blood Analysis

Blood samples were collected by cardiac puncture under carbon dioxide gas anesthesia. Blood cell counts and serum EPO concentration were determined at the end of the trial (day 21). Whole blood was collected in vacutainer tubes (Becton Dickinson, Franklin Lakes, NJ, USA) coated with ethylenediaminetetraacetic acid (EDTA) anticoagulant. White blood cell (WBC), lymphocyte, monocyte, neutrophil, eosinophil, basophil, RBC, hemoglobin (Hb), hematocrit (Hct), mean corpuscular volume (MCV), mean corpuscular hemoglobin (MCH), and platelets (PLT) concentrations were measured using an automatic blood cell counter (Cell counter analyzer, MS9-5V; Melet Schloesing Laboratoires, Osny, France). Whole blood samples were analyzed within 5 h. The remaining blood was collected in plain vacutainer tube (Becton Dickinson, Franklin Lakes, NJ, USA), and the serum separated by centrifugation (4000 rpm, 10 min). Serum EPO was measured using a rat EPO enzyme-linked immunosorbent assay (ELISA) kit (Cusabio, Houston, TX, USA). All serum samples were stored at −80 °C until analysis. Serum creatinine was determined using the hexokinase enzyme method using an AU680 automated chemistry analyzer (Beckman Coulter Inc., Pasadena, CA, USA).

### 2.3. Quantitative Real-Time Polymerase Chain Reaction and Data Analysis in Rat Kidney 

Total ribonucleic acid (RNA) was extracted using Trizol reagent (Gibco BRL, Gaithersburg, MD, USA) according to the protocol of the manufacturer. Approximately 10 mg of renal cortex tissue was thawed and homogenized in 1 mL Trizol reagent. Concentration of RNA was quantified with a Nanodrop spectrophotometer (DS-11+; DeNovix Inc., Wilmington, DE, USA). Next, cDNA was prepared using a cDNA synthesis kit (Thermo Fisher Scientific, Waltham, MA, USA). Quantitative real-time polymerase chain reaction (qPCR) was performed using a RT-qPCR kit (Thermo Fisher Scientific, Waltham, MA, USA) and a StepOne Plus Real-time PCR system (Thermo Fisher Scientific, Waltham, MA, USA). Primer specific sequences are listed in Table 1. Reactions were preheated for 10 min at 95 °C followed by 40 cycles at 95 °C for 20 s, 60 °C for 20 s, and 72 °C for 20 s. qPCR data were quantified based on the number of cycles needed for amplification-generated fluorescence to reach a specific threshold of detection (Ct value). Relative gene expression was quantified on the basis of equal amounts of RNA (1 μg) and average Ct values for each gene. Delta Ct (ΔCt = Ct_target gene_ − Ct_reference gene_) was calculated using Ct values for genes in the same sample. Actin was used as the internal reference control gene. ΔΔCt value was calculated with the following equation: ΔΔCt = (ΔCt_treated_ − ΔCt_untreated_). Normalized expression change was expressed as 2^−ΔΔCt^ (actin control was set to 1) [30].

### 2.4. Histological Analysis

Kidney tissue was surgically removed, fixed in 10% paraformaldehyde, and then 5 μm paraffin sections were prepared. Hematoxylin–eosin (H&E) staining was used to observe general histopathological changes in tissue. Kidney biopsies were fixed in 4% paraformaldehyde (Sigma–Aldrich, St. Louis, MO, USA) for immunohistochemistry. Paraffin-embedded 3-µm-thick sections were cut, mounted on POLYSINE Slides (Thermo Fisher Scientific, Waltham, MA, USA), de-waxed in xylene, and then gradually dehydrated in 70%, 80%, 90%, and 100% ethanol for 5 min each. Antigen retrieval was performed by boiling in sodium citrate buffer (10 mM sodium citrate and 0.05% Tween 20, pH 6.0). Sections were blocked for 30 min with phosphate buffered saline (PBS) containing 1% horse serum (Sigma-Aldrich, St. Louis, MO, USA), and then incubated overnight at 4 °C with anti-Epo (1:200; rabbit polyclonal IgG N-19, sc-1310) and anti-Epor (1:200; goat polyclonal IgG H-194, sc-5624) (Santa Cruz Biotechnology, Dallas, TX, USA) antibodies. For Hif immunohistochemistry, anti-Hif-1α (1:200; rabbit polyclonal IgG, NB100-479) and anti-Hif-2α (1:100; rabbit polyclonal IgG, NB100-122) (Novus Biological, Centennial, CO, USA) were used as primary antibodies. Sections were rinsed three times in PBS and incubated for 1 h at room temperature with Alexa Fluor 488-conjugated goat anti-rabbit IgG (H+L) (1:1000; A27034) and Alexa Fluor 594-conjugated rabbit anti-goat IgG (H+L) secondary antibodies (1:1000; A27016) (Invitrogen, Carlsbad, CA, USA) for anti-Epo and anti-Epor, respectively. Goat anti-rabbit IgG (H+L) Alexa Fluor 488-conjugated antibody (1:200; A-11008) (Thermo Fisher Scientific, Waltham, MA, USA) was used as a secondary antibody for anti-Hif-1α and anti-Hif-2α. After further rinsing, nuclei were counterstained with 4′,6-diamidino-2-phenylindole (DAPI) (1:10,000, Thermo Fisher Scientific, Waltham, MA, USA) in PBS. Images were captured using a confocal microscope with a FV1000 Fluoview camera (Olympus, Tokyo, Japan). Relative intensity was measured using ImageJ.

### 2.5. RNA Microarray

Total RNA was extracted using Trizol reagent (Gibco BRL, Gaithersburg, MD, USA) according to the manufacturer’s protocol. Dissolved RNA was further treated with RNase-free DNase to digest contaminated genomic DNA, and then purified using Qiaquick Rneasy Mini kit (Qiagen, Valencia, CA, USA) according to the manufacturer’s instructions. Eluted RNA was quantified using a ND-1000 spectrophotometer (NanoDrop Technologies, Inc., Wilmington, DE, USA). RNA quality was verified using 1% agarose denaturing gels and an Agilent 2100 Bio-analyzer (Agilent Technologies, Santa Clara, CA, USA). For control and test RNAs, synthesis of target cRNA probes and hybridization were performed using Agilent’s Low RNA Input Linear Amplification kit (Agilent Technologies, Santa Clara, CA, USA) according to the manufacturer’s instructions. Amplified and labeled cRNA was purified on RNase mini columns (Qiagen, Valencia, CA, USA) according to the manufacturer’s protocol. Labeled cRNA targets were quantified using a ND-1000 spectrophotometer (NanoDrop Technologies, Inc., Wilmington, DE, USA). Arrays were hybridized at 65 °C for 17 h using an Agilent Hybridization oven (Agilent Technologies, Santa Clara, CA, USA). Hybridized microarrays were washed using the manufacturer’s washing protocol (Agilent Technologies, Santa Clara, CA, USA). Fluorescence images of the hybridized arrays were generated using the Agilent DNA Microarray Scanner, and intensities were extracted using Agilent Feature Extraction software ver. 10.7.3.1. Average fluorescence intensity was calculated for each spot and the local background subtracted. All data normalization and selection of fold-changed genes were performed using GeneSpring GX 7.3.1 (Agilent Technologies, Santa Clara, CA, USA). Differentially expressed genes were identified using a single *t*-test that assumes unequal variance between groups. The criterion for identification of significant genes was a value of *p* < 0.05. RNA sequencing data have been deposited in NCBI’s Gene Expression Omnibus (GEO) database (accession code: GSE146299). For pie char and Venn diagram, the ExDEGA software (Excel based Differentially Expressed Gene Analysis, ebiogen, Seoul, Korea), which is an analysis tool that facilitates the analysis of numerous data from microarray according to classified Gene Ontology (GO) terms, was used. The genes with a *p*-value < 0.05 and a fold change of 2.0 compared with the negative control were defined as significantly changed genes.

### 2.6. Two-Dimensional Electrophoresis

Isolated kidney tissue was directly homogenized using a motor-driven homogenizer (PowerGen 125; Fisher Scientific, Hampton, NH, USA) in sample lysis solution composed of 7 M urea and 2 M thiourea containing 4% (*w*/*v*) 3-3[(3-cholamidopropyl) dimethylammoniol]-1-benzamidine. Proteins were extracted for 1 h at room temperature by vortexing. After centrifugation at 15,000× *g* for 1 h at 15 °C, insoluble material was discarded, and the soluble fraction used for two-dimensional gel electrophoresis (2DE) protein concentration was assayed by the Bradford method [31]. Immobilized pH gradient (IPG) dry strips (4–10 NL IPG, 24 cm; Genomine, Pohang, Korea) were equilibrated for 12 to 16 h with 7 M urea and 2 M thiourea containing 2% 3-[(3-cholamidopropyl) dimethylammonio]-1-propanesulfonate (CHAPS), 1% dithiothreitol (DTT), and 1% pharmalyte, and loaded with 200 μg of protein. Isoelectric focusing (IEF) was performed at 20 °C using a Multiphor II electrophoresis unit and EPS 3500 XL power supply (Amersham Biosciences, Piscataway, NJ, USA), following the instructions of the manufacturer. During IEF, the voltage was linearly increased over 3 h from 150 to 3500 V for sample entry, followed by a constant 3500 V, with focusing complete after 96 kVh. Prior to the second dimension, strips were incubated for 10 min in equilibration buffer (50 mM Tris-Cl, pH 6.8 containing 6 M urea, 2% sodium dodecyl sulfate (SDS), and 30% glycerol), first with 1% DTT and second with 2.5% iodoacetamide. Equilibrated strips were inserted onto sodium dodecyl sulphate–polyacrylamide gel electrophoresis (SDS-PAGE) gels (20 × 24 cm, 10%–16%). SDS-PAGE was performed using the Hoefer DALT 2DE system (Amersham Bioscience, Piscataway, NJ, USA) following the instructions of the manufacturer. 2DE gels were run at 20 °C for 1700 Vh, and then silver stained (but without fixing and glutaraldehyde sensitization). Quantitative analysis of digitized images was performed using PDQuest software (version 7.0; Bio-Rad, Hercules, CA, USA), according to the protocols of the manufacturer. The quantity of each spot was normalized by total valid spot intensity. Protein spots expressed over two-fold in expression levels compared with controls from the normal sample were selected. The NCBInr database was searched using Mascot software, and identified spots were mapped onto gels stained with Coomassie brilliant blue. For protein identification, protein spots were excised, digested with trypsin (Promega, Madison, WI, USA), mixed with α-cyano-4-hydroxycinnamic acid (CHCA) in 50% acetonitrile (ACN)/0.1% trifluoroacetic acid (TFA), then subjected to matrix assisted laser desorption ionization–time of flight–mass spectrometry (MALDI-TOF-MS) analysis (Ettan MALDI-TOF; Amersham Bioscience, Piscataway, NJ, USA), as described previously [32]. 

### 2.7. Protein Data Bioinformatics Analysis

Differentially expressed proteins were subjected to GO analysis, pathway enrichment, and protein–protein interaction (PPI) analysis. GO analysis was performed using the Database for Annotation, Visualization and Integrated Discovery (DAVID) and included biological processes (BP), cell components (CC), and molecular functions (MF). Pathway enrichment was performed using the Kyoto Encyclopedia of Genes and Genomes (KEGG) database, with significant pathways analyzed. Based on interactions among pathways in the KEGG database, PPI were built using the Search Tool for Retrieval of Interacting Genes/Protein (STRING) database. 

### 2.8. ATP Measurement and NADH Dehydrogenase Assay in Mitochondria

Intact mitochondria were immediately isolated from the kidney by differential centrifugation using a Mitochondria Isolation kit (Thermo Fisher Scientific, Waltham, MA, USA). The pellet was re-suspended in PBS, and freshly isolated mitochondria immediately used for analysis of ATP content. Mitochondrial protein concentration was determined using the Bradford method. Mitochondrial ATP content was measured by a luminescence assay using the ATPlite ATP detection system (Perkin-Elmer, Waltham, MA, USA). For the NADH dehydrogenase activity assay, mitochondria were isolated, as described above, with contamination from cytosolic dehydrogenase prevented. Mitochondrial NADH dehydrogenase activity was measured using a mitochondrial complex I activity assay kit (BioVision, Milpitas, CA, USA).

### 2.9. Statistical Analysis

Statistical analysis was performed with SPSS 12.0 (IBM Corporation, Armonk, NY, USA). Results were expressed as mean ± standard deviation (SD) of three independent experiments. Statistical significance of differences was determined by Student’s *t*-test. A level of *p* < 0.05 was considered significant.

## 3. Results

### 3.1. Growth Performance

The effect of GABA supplementation on weight gain, food intake, and food conversion rate was investigated in rats that initially weighed an average of 175 g (Table 2). Rats consumed 17.6 to 18.3 g of diet per day and gained 117.9 to 119.6 g of body weight in 3 weeks. There were no significant differences in these parameters among groups.

### 3.2. Serum Erythropoietin

EPO levels in GABA-treated groups significantly increased compared with negative controls (Table 3). Administration of GABA for 3 weeks at concentrations of 50, 100, and 200 ppm showed a significant increase in EPO levels (97.2 ± 10.7, 97.7 ± 11.2, and 120.1 ± 14.9 mIU/mL, respectively) compared with the negative control (59.9 ± 10.3 mIU/mL) in a dose-dependent manner. In particular, EPO levels increased 2.01-fold in rats treated with 200 ppm GABA compared with the negative control.

### 3.3. Total and Differential White Blood Cell Counts

To investigate the effect of GABA on WBC, differential WBC counts were evaluated. Differential WBC counts in GABA-treated rats were not significantly different compared with the negative control in all experimental groups (Table 4).

### 3.4. Red Blood Cell Indices

Hematological values were measured in whole blood to investigate of the effect of GABA on normal hematological parameters. Although RBC count increased in the G50 and G200 groups, no significant differences were found in RBC indices (including MCV, HCT, MCH, MCHC, Hb, and PLT from any groups (Table 5). Unexpectedly, Hb was not increased by GABA. In rats treated with 200 ppm GABA, RBC were significantly increased (6.3 ± 0.28 × 10^6^/mm^3^) (*P* = 0.038) compared with the negative control (5.9 ± 0.23 × 10^6^/mm^3^). This suggests that GABA (200 ppm) might contribute to improved anemia.

### 3.5. Expression of EPO-Related Genes and Proteins by GABA

**Figure 1:**
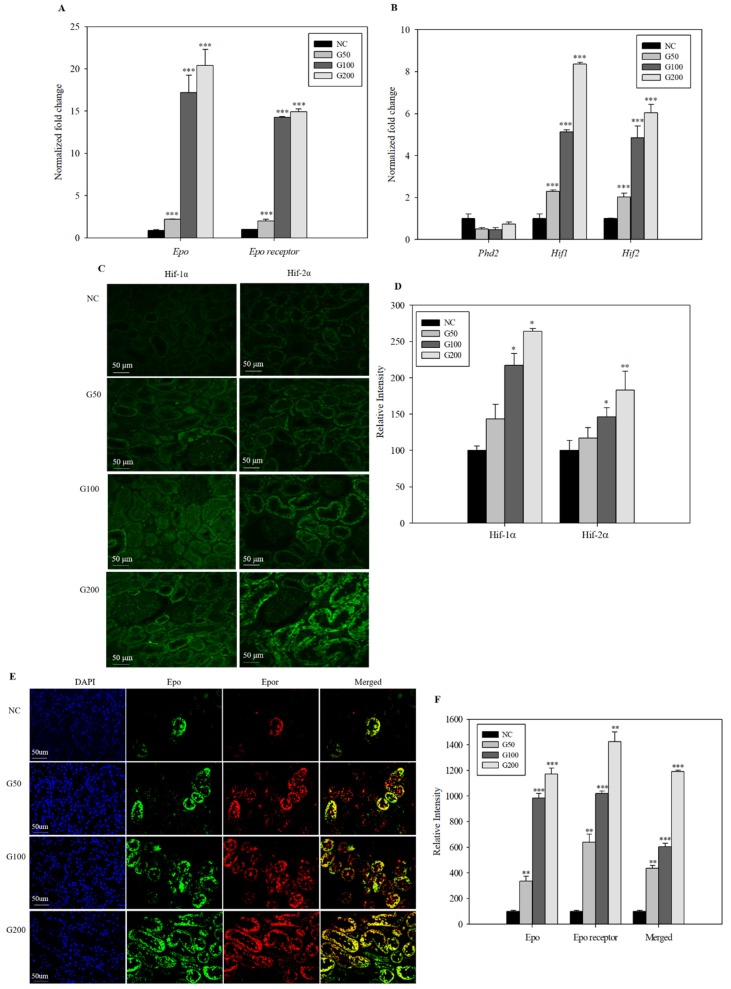
Expression levels of erythropoiesis-related genes and Epo and Epor protein immunofluorescence in GABA-treated rat kidney. (**A**) Gene expression of *Epo* and *Epor*. (**B**) Gene expression of *Phd2*, *Hif-1α*, and *Hif-2α.* Data were normalized using actin as a control. Results are expressed as mean ± SD of three independent experiments, * *p* < 0.05, ** *p* < 0.01, and *** *p* < 0.001, Student’s *t*-test, compared with the negative control. (**C**) Paraffin sections were fixed and stained with anti-Hif-1α and Hif-2α antibodies. The images are the representative of the eight slides of each group. All bars are 50 μm (40×). (**D**) Relative intensity of Hif-1α and Hif-2α protein production in rats treated with GABA at concentrations of 50, 100, and 200 ppm. (**E**) Paraffin sections were fixed and stained with anti-Epo and anti-Epor antibodies. The images are the representative of the eight slides of each group. All bars are 50 μm (40×). (**F**) Relative intensity of Epo and Epor protein production in rats treated with GABA at concentrations of 50, 100, and 200 ppm.

The transcription factors, Hif-1α and Hif-2α, are constitutively expressed in kidney. Expression levels of *Epo*, *Epor*, *Hif-1α*, *Hif-2α*, and prolyl-hydroxylase domain protein-2α (*Phd-2α*) were measured by qPCR. Gene expression levels of *Epo*, *Epor*, *Hif-1α*, and *Hif-2α* (but not *Phd-2α*) increased in a dose-dependent manner in GABA-treated groups compared with the negative control (Figure 1A,B). *Epo* mRNA levels in rats treated with GABA (50, 100, and 200 ppm) increased 2.19-, 17.21-, and 20.42-fold, respectively, compared with the negative control. *Epor* mRNA levels increased 2.00-, 14.27-, and 14.92-fold, respectively, while *Hif-1α* mRNA levels increased 2.28-, 5.08-, and 8.28-fold, respectively, and *Hif-2*α mRNA levels increased 2.01-, 4.80-, and 5.98-fold in rats treated with GABA at concentrations of 50, 100, and 200 ppm, respectively, compared with the negative control. These results suggest that GABA activates erythropoiesis by stimulating expression of *Epo*, *Epor*, *Hif-1α*, and *Hif-2α* genes. Protein expression levels of Hif-1α and Hif-2α significantly increased in 100 and 200 ppm GABA-treated groups compared with the negative control (Figure 1C,D). Hif-1α levels increased 1.43-, 2.17-, and 2.64-fold, respectively, and Hif-2α levels increased 1.16-, 1.46-, and 1.83-fold in rats treated with GABA at concentrations of 50, 100, and 200 ppm, respectively, compared with the negative control. The results show that Hif-1α and Hif-2α increase by GABA supplementation in transcriptional and translational levels.

### 3.6. Effect of GABA on Epo and Epo Receptor Production

Production of Epo localized with Epor was evaluated in the kidney by confocal microscopy. Double staining showed increased expression levels of Epo and Epor in rats treated with GABA compared with the negative control. Further, Epo was in close proximity to Epor expressed in kidney cells (Figure 1E). In rats treated with GABA at concentrations of 50, 100, and 200 ppm, relative intensity of Epo protein labeling was 336.2 ± 200.0%, 984.4 ± 190.7%, and 1171.9 ± 248.3%, respectively, compared with the negative control (100 ± 64.4%). While in rats treated with GABA at concentrations of 50, 100, and 200 ppm, relative intensity of Epor protein labeling was 639.6 ± 226.7%, 1020.8 ± 170.4%, and 1424.3 ± 453.7%, respectively, compared with the negative control (100 ± 46.2%) (Figure 1F). Expression levels of Epo and Epor were consistent with gene expression. Although it is known that Epo is produced in interstitial fibroblasts closely connected with the proximal tubule in the kidney, in some case Epo immunopositivity was observed in proximal tubules [33,34]. In this study, Epo stained in the renal proximal tubules as well.

### 3.7. Serum Creatinine

To evaluate the toxicity of GABA, serum creatinine was measure. In all groups, serum creatinine levels were not significantly different, which means that GABA was not toxic to rats (Table 6).

### 3.8. Histological Analysis of Kidneys

**Figure 2:**
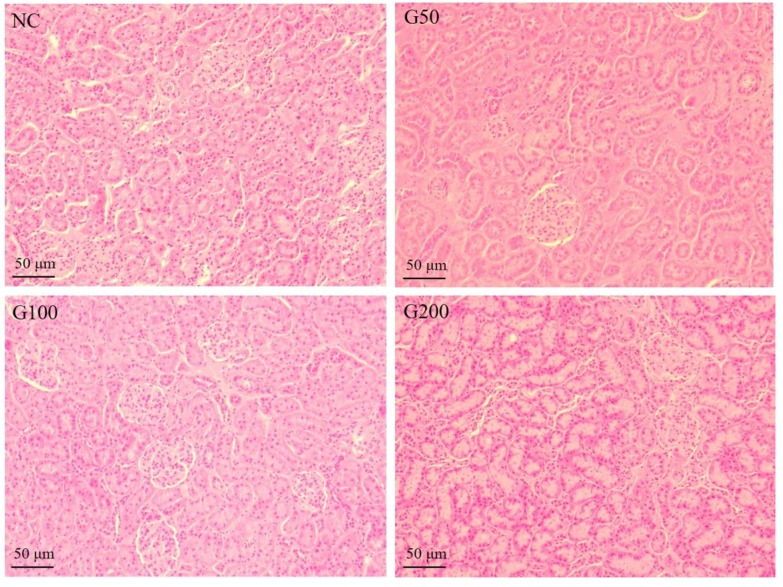
Hematoxylin and eosin (H&E) staining of GABA-treated rat kidney. There are no significant changes of cell morphology in GABA-fed rat kidney tissues. All bars are 50 μm (Magnification 40×).

To examine kidney cytotoxicity of GABA, histological analysis was performed on kidney tissue from experimental rats. In all groups, the kidney was free from any pathological abnormality, and stained sections showed a normal appearance with regular cellular structure (Figure 2). 

### 3.9. Gene Expression Analysis of Kidney Tissue in GABA-Treated Rats

**Figure 3:**
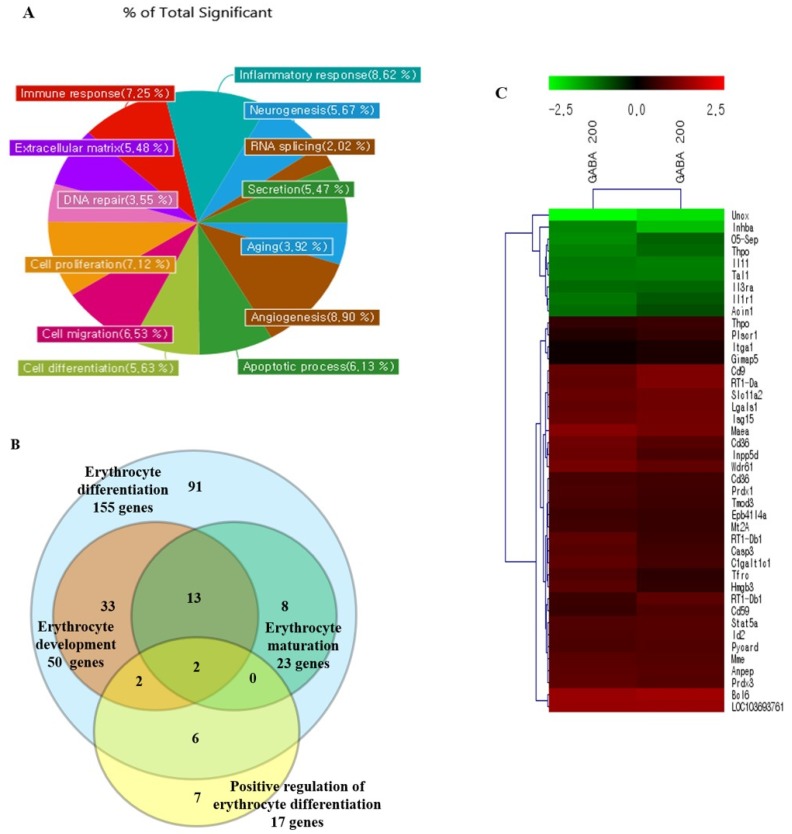
Differential gene expression between normal and 200 ppm GABA-treated rat kidneys based on RNA microarray data. (**A**) Pie chart representation of GO for genes differentially expressed in microarray analyses and summarized according to major categories. Gene expression in GABA-treated kidney tissue was compared to normal tissue, and the criterion for differential expression was the 2-fold change (*p* < 0.05 of the DEGs). Thirteen of major functional categories were obtained using GO annotation. (**B**) A Venn diagram displaying overlap of significant genes found in the erythrocyte differentiation, maturation, and development. Gene numbers were obtained by GO annotation generated by 2-fold, *p* < 0.05 criterion. (**C**) Heat map of rat orthologous genes involved in erythropoiesis and hematopoiesis that show a significant difference in gene expression (false discovery rate, FDR < 0.05). Each row represents one gene and each column represents two samples (two kidney tissues from 200 ppm GABA group). Red and green indicate over- and under-expressed genes, respectively. Expression levels are proportional to the brightness of color (see color bar). Black indicates no difference in expression level between normal rat kidney and G200 kidney tissue.

The gene expression profile of kidney tissue in GABA-treated rats was evaluated by RNA microarray analysis and compared with the negative control. From 3025 genes analyzed by RNA microarray, genes were identified that were significantly differentially expressed in the kidney of 200 ppm GABA-treated rats compared with the negative control. Of the 3025 genes, 758 genes showed >2-fold higher or lower expression levels. Among these, 550 genes were down-regulated (<0.5-fold) and 208 genes were up-regulated (>2-fold) in the kidney of 200 ppm GABA-treated rats compared with the negative control. GO analysis showed 13 major functional categories based on the criterion differently expressed in microarray analysis (2-fold change, *p* < 0.05 of the DEGs) (Figure 3A). Total of 162 genes differently expressed were involved in erythrocyte differentiation, development, and maturation, and among them, 17 genes were involved in the positive regulation of erythrocyte differentiation (Figure 3B). Among the genes selected for hierarchical cluster analysis, 25 genes were erythropoiesis-related genes and 16 genes were hematopoiesis-related genes, with approximately 73.2% of the total 41 genes showing increased expression levels (Figure 3C). 

### 3.10. A Significant Expression of The Proteins Involved in The Citric Acid Cycle in GABA-Treated Rat Kidney

**Figure 4:**
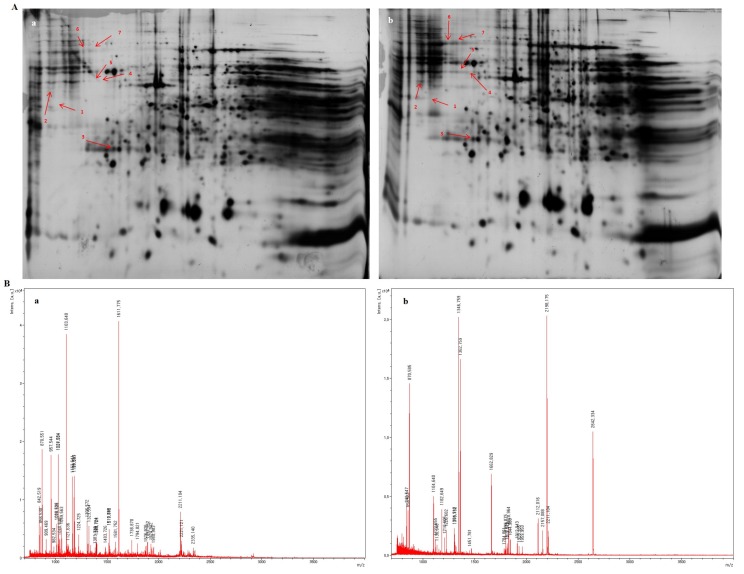
Two-dimensional gel electrophoresis of normal and 200 ppm GABA-treated rat kidneys. (**A**) The full-length gels are representative two-dimensional protein electrophoresis of rat kidney tissue in a control group (a) and G200 group (b). Soluble proteins separated using a linear pH 3.5–10 gradient in 10% to 16% SDS-PAGE gels were stained with Coomassie G250, digitalized, and compared using image analysis software. Red symbols indicate up-regulated proteins (Table 7). (**B**) Representative MALDI-TOF-MS maps. (a) Peptide mass fingerprinting (PMF) of NADH dehydrogenase 1 alpha and (b) PMF of DOPA decarboxylase.

2DE gel separation of proteins from rat kidney treated or untreated with GABA was performed. For preparation of 2DE maps, each sample was analyzed separately without pooling, with attempts to minimize the influence of methodology for each experiment. Three kidney tissues from control and experimental groups were screened for significant differences in expression. Representative 2DE gel images are shown in Figure 4A. Seven differentially expressed proteins were evaluated by MALDI-TOF-MS analysis. The results are summarized in Table 7. A probability-based score of each identified protein was considered significant (*p* < 0.05). Among them, protein expression levels of spots 1 and 2 showed a significant increase to 239.3- and 259.6-fold, respectively. Spots 1 and 2 were identified as nicotinamide adenine dinucleotide (NADH) dehydrogenase (GI number 564349298) and dihydroxyphenylalanine (DOPA) decarboxylase (GI number 149016961), respectively. Their representative MALDI-TOF-MS maps are shown in Figure 4B. The results of protein profiles suggested that the citric acid cycle was activated by GABA treatment, which might induce the oxidation of NADH resulted in the increase of ATP level.

### 3.11. Gene Ontology Enrichment and Protein–Protein Interaction Analysis

**Figure 5:**
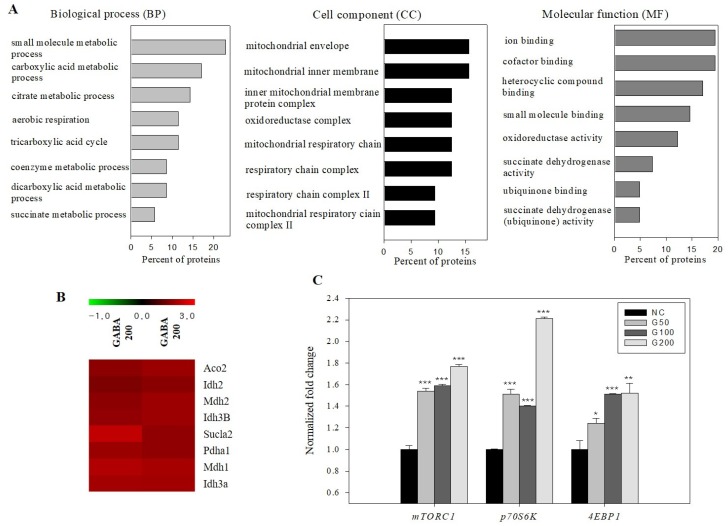
Gene ontology classification of proteins differentially expressed between normal and 200 ppm GABA-treated rat kidney. Screening of RNA microarray results for genes associated with the citrate cycle, and gene expression associated with an EPO mechanism. (**A**) Differentially expressed proteins were grouped into three hierarchically structured GO terms: biological process (BP), cellular component (CC), and molecular function (MF). (**B**) Heat map showing rat orthologous genes related to the citrate cycle and with a significant difference in gene expression (FDR < 0.05). Each row represents one gene and each column represents two samples (two kidney tissues of 200 ppm GABA group). (**C**) Gene expression of *mTORC*, *p70S6K*, and *4EBP1*. Data were normalized using actin as a control. Results are expressed as mean ± SD of three independent experiments, * *p* < 0.05, ** *p* < 0.01, and *** *p* < 0.001, Student’s *t*-test, compared with the negative control.

The seven differentially expressed proteins identified by Mascot analysis were subjected to GO. Based on the terms represented in the GO database, the differentially expressed proteins were divided into three categories: BP, CC, and MF. The top eight enriched BP, CC, and MF from the 200 ppm GABA-treated group compared with the control group are shown in Figure 5A. BP analysis revealed that most identified proteins were distributed in the small molecule metabolic process, carboxylic acid metabolic process, citrate metabolic process, aerobic respiration, and tricarboxylic acid (TCA) cycle. The top CC were mitochondrial envelopment, mitochondrial inner membrane, and inner mitochondrial membrane protein complex. The three functions with highest significance levels in MF were ion binding, cofactor binding, and heterocyclic compound binding. Among the genes analyzed by RNA microarray, TCA cycle-related genes were up-regulated. Comprehensive analysis of BP, CC, and MF results showed that the TCA cycle was the most important point (Figure 5B).

To determine the mechanism for the TCA cycle increase due to the effect of GABA in kidney tissue, gene expression of mechanistic target of rapamycin complex 1 (*mTORC1*), ribosomal protein S6 kinase beta-1 (*p70s6k*), and eukaryotic translation inhibition factor 4E-binding protein (*4EBP1*) was analyzed. *mTORC1* mRNA levels increased 1.54-, 1.59-, and 1.77-fold, *p70s6k* mRNA levels increased 1.51-, 1.40-, and 2.21-fold, and *4EBP1* mRNA levels increased 1.24-, 1.51-, and 1.52-fold in rats treated with GABA at 50, 100, and 200 ppm, respectively, compared with the negative control (Figure 5C).

**Figure 6:**
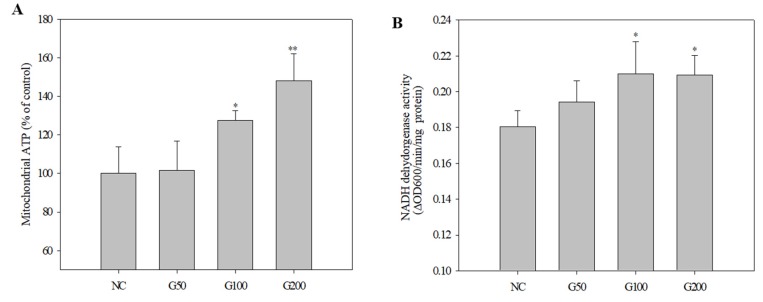
Effect of GABA on ATP production and NADH dehydrogenase activity from kidney mitochondria. (**A**) ATP quantification assay. (**B**) NADH dehydrogenase activity assay. Results are expressed as mean ± SD of three independent experiments, * *p* < 0.05 and ** *p* < 0.01, Student’s *t*-test, compared with the negative control.

Further analysis of biological pathways showed that 10 KEGG pathways were enriched in the dataset of differentially expressed genes (Table 8). These KEGG pathways (*p* < 0.05) included the TCA cycle, metabolic pathways, carbon metabolism, oxidative phosphorylation, Parkinson’s disease, non-alcoholic fatty acid liver disease (NAFLD), Alzheimer’s disease, Huntington’s disease, microbial metabolism in diverse environments, and biosynthesis of amino acids. Many of these metabolic pathways were changed in rat kidney treated with GABA. The four key changed pathways were the citrate cycle, metabolism pathways, carbon metabolism, and oxidative phosphorylation, all belonging to the metabolism classification. The most significant KEGG pathway derived by PPI analysis was the citrate cycle. Consequently, we measured ATP production to examine the increase in citrate cycle activity in GABA-treated rat kidney tissue. ATP levels in kidney treated with GABA (50, 100, and 200 ppm) increased 1.0 ± 0.15%, 27.6 ± 0.05%, and 48.1 ± 0.14%, respectively, compared with the negative control in a dose-dependent manner (Figure 6A).

Subsequently, we analyzed the activity of NADH dehydrogenase, which showed the greatest increase in protein expression. Activity of NADH dehydrogenase in the control, G50, G100, and G200 groups was: 0.18 ± 0.009, 0.19 ± 0.012, 0.21 ± 0.018, and 0.21 ± 0.011 ΔOD_600_/min/mg protein, respectively (Figure 6B).

**Figure 7:**
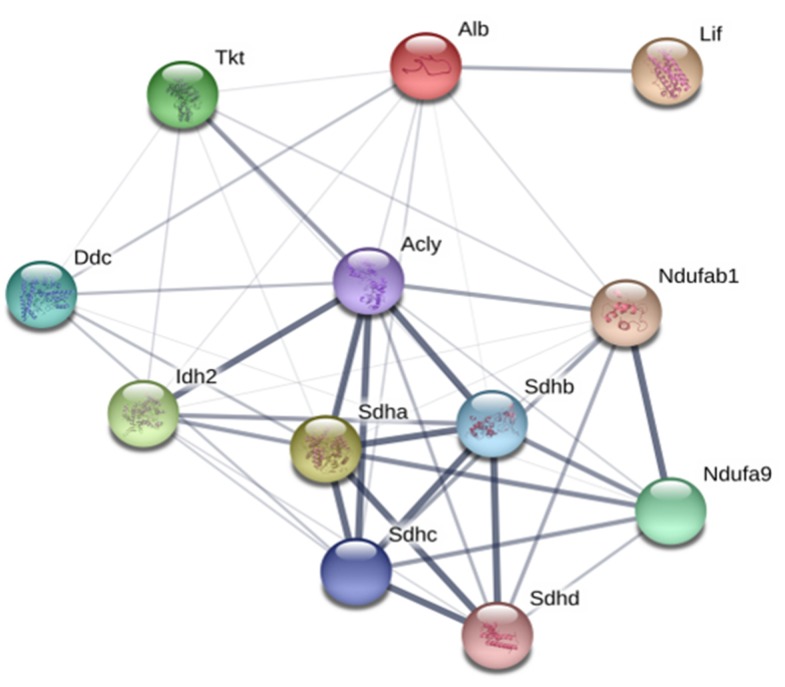
STRING analysis of protein interaction networks. Interactions of identified proteins were mapped by searching the STRING database version 10.5, with a confidence cutoff of 0.15. In the resulting protein association network, proteins are presented as nodes connected by lines whose thickness represents the confidence level.

PPI analysis examined the interaction networks affected by 200 ppm GABA in rat kidney. STRING is a meta-database program that generates a network of protein interactions from high-throughput experimental data and predictions based on genomic context analysis. The indicated node represents the protein, and the thickness of the connected lines reflects the degree of correlation between the proteins. The seven protein spots (Table 7) identified by 2DE analysis and five maximum interacting proteins are shown based on the STRING database (Figure 7). In addition to the seven proteins identified by 2DE analysis, ATP citrate lyase (Acyl) was included in the protein interaction network by STRING analysis. Acyl is the major enzyme responsible for synthesis of cytoplasmic acetyl-CoA in many tissues. It catalyzes formation of acetyl-CoA and oxaloacetate from citric acid and CoA during TCA cycle operation to promote hydrolysis of ATP, ADP, and phosphate.

## 4. Discussion

The beneficial effects of GABA have been studied, and include lowering blood pressure, improving immunity, relieving anxiety, and stroke prevention [35,36,37,38]. Human recombinant EPO has one of the world’s top ten biopharmaceutical sales. Extensive research to develop erythropoiesis-stimulating agents and to produce biosimilar EPOs for anemia treatment has been studied [39]. However, human recombinant EPO and exogenous EPO cannot be universal remedies because of their expense. Therefore, alternative strategies for EPO production in the kidney are promising methods for improving anemia. In this study, GABA stimulated EPO production in the rat kidney, with levels of serum EPO increasing in a dose-dependent manner. 

Hif-1α is degraded at normal oxygen concentrations by binding of a hydroxyl group to the proline residue in the oxygen-dependent degradation domain (ODDD) by prolyl hydroxylase (Phd). This ultimately allows for recognition and ubiquitination by the von Hippel-Lindau protein (pVhl) and E3 ubiquitin ligase complex [40,41,42]. When Phd loses its activity in a hypoxic state, *Hif-1α* is stabilized and the gene transcribed for adaptation to hypoxia [43]. In addition, Hif-2α is involved in erythropoiesis of adults and plays a different physiological role than Hif-1α [44]. In this study, the *Hif-1α* gene induced in only the hypoxic state was significantly increased after GABA intake, even in the normal oxygen state, compared with the control group. In addition, there was no significant difference in *Phd-2α* gene expression, which should be increased in normal oxygen conditions. Moreover, the *Hif-2α* gene is directly involved in erythropoiesis and was significantly increased after GABA intake. This suggests that GABA does not directly affect Phd to stabilize Hif-1α, instead GABA might directly affect Hif expression. 

In this study, although RBC count in G100 group did not show a significant increase compared with the control group, RBC count showed a tendency to increase in the GABA-administered groups. Enhancement of RBC production is caused by a homeostatic mechanism of higher organisms [45]. In addition, GABA increased EPO level but not Hb level. In a previous report, the administration of 5000 IU human EPO (rHuEpo) for 15 weeks in 8 healthy male volunteers significantly increased Hb after 8 weeks by increasing red blood cell volume and decreasing plasma volume [46]. The recommended initial dose of epoetin alfa (rHuEpo) for chronic kidney disease adult patients is 50 to 100 U/kg three times weekly, intravenously or subcutaneously [47], which have been approved by the U.S. Food and Drug Administration (FDA). Although the exact equivalent dose cannot be converted between human and rat, in this study, the increased concentration of EPO by GABA (50 to 200 ppm) treatment was 37 to 60 mIU, which might be not enough to significantly increase Hb in non-anemic rat within the experimental period. In this study, we used non-anemic animal model, which may be another reason why Hb level did not increase, even though EPO increased by GABA administration. Further studies are needed on the improvement effect of GABA on anemia in animal model of anemia.

GABA is approved and generally recognized as safe (GRAS) by the Food and Drug Administration (FDA), and is used as a food supplement [48]. Kidney tissue from each group was examined, with H&E staining performed to determine kidney toxicity. No clinical symptoms such as tissue necrosis or inflammation were observed, and it was confirmed that ingestion of GABA does not act as a toxin in rats. Serum creatinine level suggested that GABA supplementation did not show toxicity as well. 

Protein expression profile analysis comparing control and GABA administration groups found seven significantly increased proteins compared with the control group, with two proteins (DOPA decarboxylase and NADH dehydrogenase) greatly increased. DOPA decarboxylase decomposes 3,4-dihydroxyphenylalanine into carbon dioxide and amines, and is involved in dopamine biosynthesis [49]. Dopamine is reported to increase in the brain when recombinant EPO is administered to mice [50]. In addition, GABA can increase or decrease dopamine turnover depending on brain area analysis, duration of stimulation, and administration of dopamine antagonist [51]. Although there may be differences in concentration from the present study, it is thought that increased EPO due to GABA may affect increased DOPA decarboxylase expression. 

NADH dehydrogenase transfers electrons from NADH to ubiquinone [52]. NADH supports ATP synthesis, which is dependent on electron route through the electron transport chain and homeostasis [53]. Excess NADH in the citrate cycle significantly increases NADH dehydrogenase expression to maintain NAD and ATP homeostasis in mitochondria. Expression of the NADH dehydrogenase gene in human vascular cells is approximately 1.4-fold increased by human recombinant EPO [54]. In the present study, NADH dehydrogenase protein was greatly increased by GABA, which may be due to differences in endogenous and exogenous EPO.

Leukemia inhibitory factor (Lif) is a remarkable polyfunctional regulator, with striking actions on a diverse range of cell types, although it has not yet been demonstrated to be a major proliferative factor for hematopoietic cells [55]. It is not apparent that Lif alone activates differentiation into normal fetal cells or adult hematopoietic cells, but when combined with interleukin-3, Lif enhances proliferation of blast colonies [56]. It is also known to be a proliferative stimulus for the continuous hematopoietic cell line, DA-1 [57]. In this study, Lif protein was greatly elevated in GABA treated rats (Table 7), which suggests that GABA might support erythropoiesis and hematopoiesis by elevating EPO and Lif. The isocitrate dehydrogenase (Idh) family of enzymes catalyzes oxidative decarboxylation of isocitrate to α-ketoglutarate and carbon dioxide. Idh plays a role in cell growth in the hypoxic condition [58]. Indeed, GABA increases Idh expression, suggesting that GABA induces a hypoxic condition. Therefore, GABA increases the activity of the TCA cycle followed by development of hypoxia resulted in the increase of expression level of Hif or vice versa, which has to be explained with intense molecular study in our further study. 

GO analysis and KEGG pathway analysis are the most reliable methods for providing greater understanding of BP, CC, and MF. In this study, overexpressed proteins were classified into different functional categories according to GO analysis. These categories included: citrate metabolic process, tricarboxylic acid cycle, mitochondrial respiratory chain complex, respiratory chain, cofactor binding, succinate dehydrogenase activity, and others (Figure 5A). KEGG pathway analysis showed that the citrate cycle, metabolic pathway, and carbon metabolism were the top three most enriched pathways (*p* < 0.05). These pathways are associated with carbohydrate metabolism, a series of chemical reactions, and carbon decomposition and synthesis. In RNA microarrays and 2DE analysis, the most significant pathway was the citrate cycle. The citrate cycle (or TCA cycle or Krebs cycle) is an important aerobic pathway for the final steps of carbohydrate and fatty acid oxidation. The cycle starts with acetyl-CoA, which is the activated form of acetate derived from glycolysis and pyruvate oxidation for carbohydrates, and from beta oxidation of fatty acids [53]. It produces ATP in mitochondria and plays a variety of roles in intracellular energy supply. 

mTORC exists as a two-cell complex with individual regulation and function. mTOR complex 1 is inhibited by the natural product rapamycin and contains a protein, raptor [59,60]. The ultimate role of mTORC1 is to regulate cell differentiation through cap-dependent translation, which affects cell size, with 4EBP1 regulating cell proliferation [61,62]. It is also reported that the mTORC1 pathway is an important regulator of RBC growth and proliferation, and that inhibiting mTORC can cause anemia [63]. Moreover, erythroid mitochondria are regulated by mTORC1-mediated protein translation and may be directly related to blood disease associated with mitochondrial dysregulation [64]. When erythrocytes are cleaved, EPO reactivity (which is an erythropoietic factor) is required, and iron is accumulated and highly proliferated [64]. Both EPO and iron activate mTORC1 and phosphorylate 4EBP1 and p70S6K to promote protein synthesis in mitochondria [63,65]. In this study, mTORC1, 4EBP1, and p70S6K expression were significantly increased in kidney tissue.

Mitochondrial stimulation activity increases cellular processes, such as Hb synthesis, by increasing ATP and cellular metabolism. Mitochondrial dysfunction is a serious sign of disease, causing erythropoiesis dysfunction. In another aspect, the balance between ATP and RBC is very important. Changes in intracellular ATP in vitro alter the shape of RBCs, and increase RBC membrane fluctuation [66,67]. RBC fluctuations are directly linked to the membrane bilayer and cytoskeletal network, which suggests that critical binding between the lipid bilayer and spectrin network is actively controlled by ATP. The results of our protein expression and KEGG pathway analysis are consistent with our gene expression and RBC production results. Altogether, these results are a reliable basis for erythropoiesis concept.

## 5. Conclusions

One of the notable findings of this study is increased expression of erythropoiesis-related genes in a hypoxic environment due to GABA intake. Expression of *Epo*, *Epor*, *Hif-1α*, and *Hif-2α* were increased, and EPO levels significantly increased in blood by GABA. By analysis of up-regulated proteins by GABA, it was found that the most significantly altered KEGG pathway was the citrate cycle, with mTORC1, p70s6k, and 4EBP1 involved in citrate cycle alteration by GABA. Increased ATP levels by GABA suggest that the citrate cycle is also activated by GABA. Consequently, GABA treatment induces a hypoxic environment by altering the energy production pathway, resulting in increased endogenous EPO levels in rats, which might contribute to preventing anemia.

## Figures and Tables

**Table 1:** Primer sequences for polymerase chain reaction (PCR) amplification.

Gene Symbol	Sense (5′–3′)	Antisense (5′–3′)
*Epo*	TACGTAGCCTCACTTCACTGCTT	GCAGAAAGTAT CG TGTGAG TGT TC
*Epor*	AGGTGGACGTGTCAGCAGGC	CGTACCTTGTGGCGTATGCAG
*Phd-2*	CCATGGTCGCCTGTTACCC	CGTACCTTGTGGCGTATGCAG
*Hif-1a*	CACTGCACAGGCCACATTCAT	AAGCAGGTCATAGGCGGTTTC
*Hif-2a*	GTCACCAGAACTTGTGC	CAAAGATGCTGTT
*mTORC1*	TTGAGGTTGCTATGACCAGAGAGAA	TTGAGGTTGCTATGACCAGAGAGAA
*p70S6K*	GGAGCCTGGGAGCCCTGATGTA	GAAGCCCTCTTTGATGCTGTCC
*4EBP1*	TAGCCCTACCAGCGATGAGCCT	GTATCAACAGAGGCACAAGGAGGTAT
*Actin*	CTTTCTACAATGAGCTGCGTG	TCATGAGGTAGTCTGTCAGG

**Table 2:** Body weight, feed intake, and feed efficiency in rats fed various concentrations of GABA for 3 weeks (*n* = 8/group).

Group	Feed Intake	Weight Gain	FCR
	(g/Head)	(g/Head)	
NC	386.3 ± 35.2	119.6 ± 11.5	3.23 ± 0.02
G50	376.7 ± 31.1	118.1 ± 17.19	3.19 ± 0.02
G100	378.6 ± 25.1	118.3 ± 12.21	3.20 ± 0.01
G200	378.5 ± 40.2	117.9 ± 26.03	3.21 ± 0.01

FCR, feed conversion ratio; NC, negative control; G50, 50 ppm GABA in water; G100, 100 ppm GABA in water; G200, 200 ppm GABA in water.

**Table 3:** Effects of GABA on serum erythropoietin in rats (*n* = 8/group).

	NC	G50	G100	G200
Erythropoietin (mIU/mL)	59.9 ± 10.3	97.2 ± 10.7 *	97.7 ± 11.2 *	120.1 ± 14.9 **

NC, negative control; G50, 50 ppm GABA; G100, 100 ppm GABA; G200; 200 ppm GABA. * *p* < 0.05 and ** *p* < 0.01, Student’s *t*-test, compared with the negative control.

**Table 4:** Total and differential white blood cell counts in rats fed various concentrations of GABA for 3 weeks (*n* = 8/group).

Group	White Blood Cell (10^3^ Cell/uL)	Lymphocyte	Monocyte	Neutrophil	Eosinophil	Basophil
		(%)	(%)	(%)	(%)	(%)
NC	11.18 ± 0.86	79.77 ± 5.25	3.49 ± 0.47	6.22 ± 0.91	2.77 ± 1.61	0.35 ± 0.07
G50	11.31 ± 1.17	79.63 ± 2.44	3.39 ± 0.71	6.60 ± 1.72	2.56 ± 1.12	0.33 ± 0.06
G100	10.89 ± 3.06	80.62 ± 4.37	3.33 ± 0.86	6.41 ± 1.40	2.88 ± 2.38	0.39 ± 0.18
G200	11.44 ± 1.54	80.06 ± 4.00	3.73 ± 0.67	6.91 ± 1.45	2.28 ± 0.90	0.38 ± 0.11

**Table 5:** The red blood cell indices in rats fed various GABA concentrations for 3 weeks (*n* = 8/group).

Group	Red Blood Cell (10^6^/mm^3^)	MCV (fl)	HCT (%)	MCH (pg)	MCHC (g/dL)	Hb (g/dL)	PLT (K/uL)
NC	5.9 ± 0.23	60.0 ± 2.57	37.2 ± 1.52	22.1 ± 0.50	36.3 ± 1.42	13.5 ± 0.25	726.6 ± 74.81
G50	6.2 ± 0.13 *	60.7 ± 1.60	37.1 ± 0.86	22.7 ± 2.07	37.7 ± 3.08	13.7 ± 0.29	757.6 ± 48.57
G100	6.2 ± 0.24	61.3 ± 1.01	37.4 ± 1.41	21.9 ± 1.12	36.1 ± 1.72	13.3 ± 0.32	679.5 ± 116.00
G200	6.3 ± 0.28 *	60.7 ± 2.33	38.2 ± 1.98	22.1 ± 0.63	37.2 ± 1.66	13.5 ± 0.31	690.3 ± 188.65

NC, negative control; G50, 50 ppm GABA; G100, 100 ppm GABA; G200, 200 ppm GABA; MCV, mean corpuscular volume; HCT, hematocrit; MCH, mean corpuscular hemoglobin; MCHC, mean corpuscular hemoglobin concentration; Hb, hemoglobin; PLT, platelet; * *p* < 0.05, Student’s *t*-test, compared with the negative control.

**Table 6:** Serum creatinine levels in rats fed various concentrations of GABA for 3 weeks (*n* = 8/group).

	NC	G50	G100	G200
Creatinine (mg/mL)	0.48 ± 0.06	0.49 ± 0.07	0.49 ± 0.06	0.49 ± 0.05

NC, negative control; G50, 50 ppm GABA; G100, 100 ppm GABA; G200; 200 ppm GABA.

**Table 7:** MS identification of differentially expressed protein spots in GABA-treated rat kidney.

Spot NO.	Protein Name	gi NO. *^a^*	Matched Peptides	Protein Sequence Coverage (%)	Protein Score	Molecular Weight (Dalton)	Expression Fold *^b^*
1	NADH dehydrogenase 1 alpha subcomplex subunit 9 (Ndufa9)	564349298	21	43	188	42,252	239.3
2	DOPA decarboxylase, isoform CRA_b (Ddc)	149016961	15	33	131	55,109	256.7
3	Leukemia inhibitory factor (Lif)	4235628	8	23	70	35,344	48.6
4	Isocitrate dehydrogenase [NADP], mitochondrial precursor (Idh2)	62079055	12	29	99	51,391	23.2
5	Serum albumin (Alb)	124028612	26	39	238	70,682	4.3
6	Transketolase isoform X1 (Tkt)	564387998	16	40	113	68,314	5.7
7	Succinate dehydrogenase, flavoprotein subunit (Sdha)	18426858	17	35	102	72,596	4.8

*^a^* gi NO.: GenInfo Identifier. *^b^* Expression fold: the fold of differently expressed proteins of the 200 ppm GABA-treated group versus the control group.

**Table 8:** Significantly enriched KEGG pathways of differentially expressed proteins.

Pathway ID	Pathway Name	Gene Symbol
20	Citrate cycle (TCA cycle)	Acly, Idh2, Sdha, Sdhb, Sdhc, Sdhd
1100	Metabolic pathways	Acly, Ddc, Idh2, Ndufa9, Ndufab1, Sdha, Sdhb, Sdhc, Sdhd, Tkt
1200	Carbon metabolism	Idh2, Sdha, Sdhb, Sdhc, Sdhd, Tkt
190	Oxidative phosphorylation	Ndufa9, Ndufab1, Sdha, Sdhb, Sdhc, Sdhd
5012	Parkinson’s disease	Ndufa9, Ndufab1, Sdha, Sdhb, Sdhc, Sdhd
4932	Non-alcoholic fatty liver disease (NAFLD)	Ndufa9, Ndufab1, Sdha, Sdhb, Sdhc, Sdhd
5010	Alzheimer’s disease	Ndufa9, Ndufab1, Sdha, Sdhb, Sdhc, Sdhd
5016	Huntington’s disease	Ndufa9, Ndufab1, Sdha, Sdhb, Sdhc, Sdhd
1120	Microbial metabolism in diverse environments	Acly, Idh2, Tkt
1230	Biosynthesis of amino acids	Idh2, Tkt
